# Genomic identification of a pair of multidrug-resistant but non-pathogenic *Salmonella enterica* serovar Goldcoast isolates in southeast China

**DOI:** 10.3389/fmicb.2025.1540843

**Published:** 2025-02-26

**Authors:** Yongjuan Yuan, Ping Li, Wei Shen, Min Li, Xiaofei He, Bin Zhou

**Affiliations:** ^1^Jiashan County Center for Disease Control and Prevention, Jiaxing, China; ^2^Jiaxing Center for Disease Control and Prevention, Jiaxing, China; ^3^Medical Research Center, The First Affiliated Hospital of Wenzhou Medical University, Wenzhou, Zhejiang Province, China

**Keywords:** *Salmonella enterica* serovar Goldcoast, multidrug resistance, *β*-Lactamase, H_2_S generation, thiosulfate reductase

## Abstract

**Introduction:**

*Salmonella* is an important foodborne pathogen that can induce severe diseases such as gastrointestinal disease and typhoid fever. Accumulating evidence revealed that *Salmonella*’s resistance to antibiotics also seriously affects human health. Pathogenic *Salmonella enterica* serovar Goldcoast (*S.* Goldcoast) was first detected in 2010 in China and was predicted to have an increasing tendency.

**Methods:**

The MacConkey agar, Salmonella Shigella agar, three-sugar iron agar slant, and Gram-stained microscopic examination were used for strain identification. Gram-negative bacteria identification cards explored more properties of the isolates, while antimicrobial susceptibility testing was used to examine the multidrug resistance. The 2nd and 3rd generation sequencing revealed the genetic information of the isolates.

**Results:**

Two non-pathogenic isolates with multidrug resistance, JS33 and JS34, harbored 42 antibiotic-resistant genes (ARGs) in contig1 and 13 ARGs in contig2, were isolated from a healthy donor living in southeast China and identified as *S.* Goldcoast (6,8:r:l,w). Interestingly, JS33 and JS34 showed identical responses to more than 20 antimicrobial agents and were resistant to ampicillin, selectrin, chloramphenicol, tetracycline, and streptomycin. However, JS33 differed from JS34 in hydrogen sulfide (H_2_S) generation. The genomic sequencing identified a deletion in thiosulfate reductase (K08352) in JS34.

**Discussion:**

H_2_S is an essential physiological regulator linked to inflammation and cancer. Therefore, genomic identification of JS33 and JS34 provided us with a better understanding of drug resistance and could be used as model strains to study the effects of microbial H_2_S production on the host. Since JS33 and JS34 did not induce gastrointestinal infection or other clinical symptoms as previously reported, the appearance of non-pathogenic *S.* Goldcoast in southeast China warned us to prepare for the prevalence of antimicrobial-resistant *S.* Goldcoast in China.

## 1 Introduction

*Salmonella* is a common foodborne pathogen, and *S. enterica* and *S. bongori* can induce severe diseases, such as gastrointestinal disease and typhoid fever (Lamichhane et al., 2024). The Disease Control and Prevention (CDC) center estimates 1.35 million infections, 26,500 hospitalizations, and 420 deaths annually, with about $3.3 billion in costs in the United States due to *Salmonella* infection (Hoffmann et al., 2012). *S. enterica* was dominant in causing human infection in China; that serovars *S. typhi* and *S. paratyphi*-A, B, or C can cause typhoid and paratyphoid fevers in humans, whereas other serovars are loosely described as non-typhoidal *Salmonella* (NTS), accounting for more than 98% of *S. enterica* isolates (Manesh et al., 2021).

*S.* Goldcoast is a NTS with high plasmid carrier rates and the cytolethal distending toxin subunit B (cdtB toxin) commonly, accounting for 2.14% frequency of serovars in *S. enterica* isolates and 2.59% of human origin in China. The trend was expected to increase according to the analysis of temporal and spatial dynamics of antimicrobial-resistant *S. enterica* from 2006 to 2019 in China (Wang et al., 2023a). Based on the extensive study, we derived the first appearance of *S.* Goldcoast in 2010 in Fujian province and its prevalence in Shanghai (Wang et al., 2023a). Few cases were reported in Zhejiang province, and all reported cases exhibited gastrointestinal infection or other clinical symptoms, independent of age and gender (Wang et al., 2023a).

Broad-spectrum antibiotics are used for bacteremia, invasive NTS infections, and disseminated typhoidal *Salmonella* infections (Smith et al., 2016; Gal-Mor et al., 2014). However, the response toward antibiotics varied depending on the bacteria’s serotype and the host’s immune response. *Salmonella*’s multidrug resistance (MDR), a global issue affecting countries at all income levels, leads to economic problems worldwide (Aleksandrowicz et al., 2023). *S.* Goldcoast showed a higher proportion of MDR rate of human origin (66.67%) than that of non-human origin (41.67%) (Wang et al., 2023a). Although the current understanding of MDR, including gene mutation, efflux pumps, passivating and inactivating enzymes encoded by drug resistance genes, and the transfer of genetic resistance gene elements in bacteria, has shed some light on the issue, the global community is still grappling with antimicrobial resistance, and further research, particularly into the underlying mechanisms of MDR in *Salmonella,* is crucial (Gaurav et al., 2023; Darby et al., 2023).

Moreover, most *Salmonella* produce hydrogen sulfide (H_2_S), a beneficial gas regulating cardiovascular activity, nerve conduction, anti-inflammation, and metabolism if properly activated (Han et al., 2022). However, the mechanisms by which H_2_S regulates various physiological functions remain unclear. In the present study, a pair of non-pathogenic *Salmonella* isolates were isolated from a healthy female and identified as *Salmonella enterica* serovar Goldcoast according to the White-Kauffmann-Le Minor antigenic table, indicating a growth of microbial diversity of *S.* Goldcoast in southeast China. Antibacterial drug sensitivity tests showed that both isolates had MDR to ampicillin, chloramphenicol, tetracycline, and streptomycin. However, their ability to generate H_2_S was quite different. Therefore, deep sequencing of these two isolates was adapted to help us understand the mechanism underlying *Salmonella*’s multidrug resistance and H_2_S generation.

## 2 Material and method

### 2.1 Reagents

Selenite Brilliant Green (SBG) enrichment solution (#HB8606, Qingdao Haibo), Blood plate (#CP10002, Shanghai Kemagar), *Salmonella Shigella* (SS) agar medium (#HB4089, Qingdao Haibo), Xylose lysine deoxycholate (XLD) agar medium (#HB4105, Qingdao Haibo), MacConkey agar medium (#HB6238-9, Qingdao Haibo), three-sugar iron agar slant (#HB4088, Qingdao Haibo), Gram staining solution (#HB8278, Qingdao Haibo), Gram-negative bacteria identification card (#21341, Merieux, France), *Salmonella* typing diagnostic serum (#882116,#152,116,#332,106, Senyan, Japan), Antimicrobial Susceptibility Testing (AST) panel for aerobic Gram-negative bacilli (#B3226B, Thermo Fisher, America), DNA extraction reagents (#51304, QIAGEN); all reagents were used within their expiry dates.

### 2.2 Equipment

Constant temperature incubator (MIR-H263L-PC, PHCBI), Optical microscope (CX21FS1, Olympus), Automatic microbial identification and drug sensitivity analysis system (VITEK 2 COMPACT, Merieux, France), Turbidimeter (DensiCHEK plus, Merieux, France), Microbial susceptibility instrument (Vizion^®^, Thermo), High-throughput sequencer (model: 550, Illumina) were used for sequencing bacterial genomes (2nd generation), etc.

### 2.3 Materials

The *S. enterica* isolates 2023JS33 and 2023JS34 were extracted from the stool sample of a healthy female, 52 years old, located in southeast China, without typhoid fever or any gastrointestinal complaints. The studies involving humans were approved by Committee of Zhejiang Provincial Center for Disease Control and Prevention. The studies were conducted in accordance with the local legislation and institutional requirements. Written informed consent for participation in this study was provided.

### 2.4 Isolation

An appropriate amount of feces was inoculated in SBG enrichment broth and incubated at 36 °C for 24 h. Then, broth containing bacteria was inoculated on SS agar medium and MacConkey agar medium by drawing lines in sections and incubated at 36 °C for 24 h. After that, a single colony was selected and inoculated with Triple Sugar Iron (TSI) and incubated at 36 °C for 24 h. Pick the interested bacterial species for microscopic examination with Gram stain and subsequently inoculate into blood plates and incubate at 36°C for 24 h. The purified bacterial species were identified by automatic biochemical identification and examined with the serum agglutination test.

### 2.5 Automatic biochemical identification

One to two single colonies were picked by inoculation rings and emulsified in sterile water. Adjust solution to 0.5 McFarland turbidity for biochemical identification with Gram-negative bacteria identification cards that had been rewarmed in advance. The sterilized saline was used as agglutination control and the agglutination phenomenon was observed within 2 min.

### 2.6 Serum agglutination test

An appropriate amount of *Salmonella* serum was dropped on a clean slide and mixed with the bacterial moss, picked out by an inoculation ring, and sterilized saline thoroughly. If no agglutination was observed, other commercial serums were used to conduct serum agglutination tests one by one, according to the instructions of reagents.

### 2.7 Antibacterial drug sensitivity test

The antimicrobial susceptibility of the isolates was determined by microdilution broth assay. In detail, tested isolates (2023JS33,2023JS34) and quality control strain (ATCC25922) were streaked and inoculated on blood agar plates and incubated at 36°C for 24 h. Individual colonies were picked with an inoculation ring seeded again on blood agar plates and incubated for 24 h at 36 °C. One or two colonies were picked from freshly prepared blood agar plates and emulsified in sterile water. Adjusted the solution to 0.5 McFarland turbidity and mixed thoroughly. Then, the bacterial suspension prepared above 10 μL was added to a test tube containing 11 mL cation-adjusted Mueller-Hinton broth (CAMHB) and mixed well. The mixture should be used within 15 min. Replace the test tube cover with a Sensititre^®^ disposable sampling head and add the sample to the CHNENF drug sensitivity test plate according to AIM^®^ instructions. Remove the test tube/sampling head combination from AIM^®^ within 30 s after completion of sample loading in the drug susceptibility plate.

After the inoculation of the drug sensitivity plate, the purity of the final culture solution was checked, and all micropores were covered with a sealing film. After the incubation at 36°C for 24 h, all samples were read with a microbial susceptibility instrument, Vizion^®^. The minimum inhibitory concentration (MIC) of the drugs that naked eye could see was recorded and defined as sensitive (S), moderately sensitive (I) and resistant (R) according to the standard of Clinical and Laboratory Standards Institute (CLSI) (2023). The quality control strain was *Escherichia coli* ATCC25922. As CLSI does not provide streptomycin resistance breakpoint, it was determined according to the National Antimicrobial Resistance Monitoring System (NARMS) MIC criteria [Centers for Disease Control and Prevention (CDC), 2018].

### 2.8 Sequencing

Two isolates (2023JS33 and 2023JS34) were sent to the genetic testing laboratory of Zhejiang Tianke High-tech Development Co., Ltd. for deep sequencing (3rd generation sequencing). Whole genomic DNA was extracted by Gentra Puregene Yeast/Bact Kit (Qiagen, Valencia, CA) and sequenced using the GridION X5 platform (Oxford Nanopore Technology).

## 3 Results

### 3.1 Identifying *Salmonella enterica* serovar Goldcoast strains with different H_2_S generation capacities

**Figure 1:**
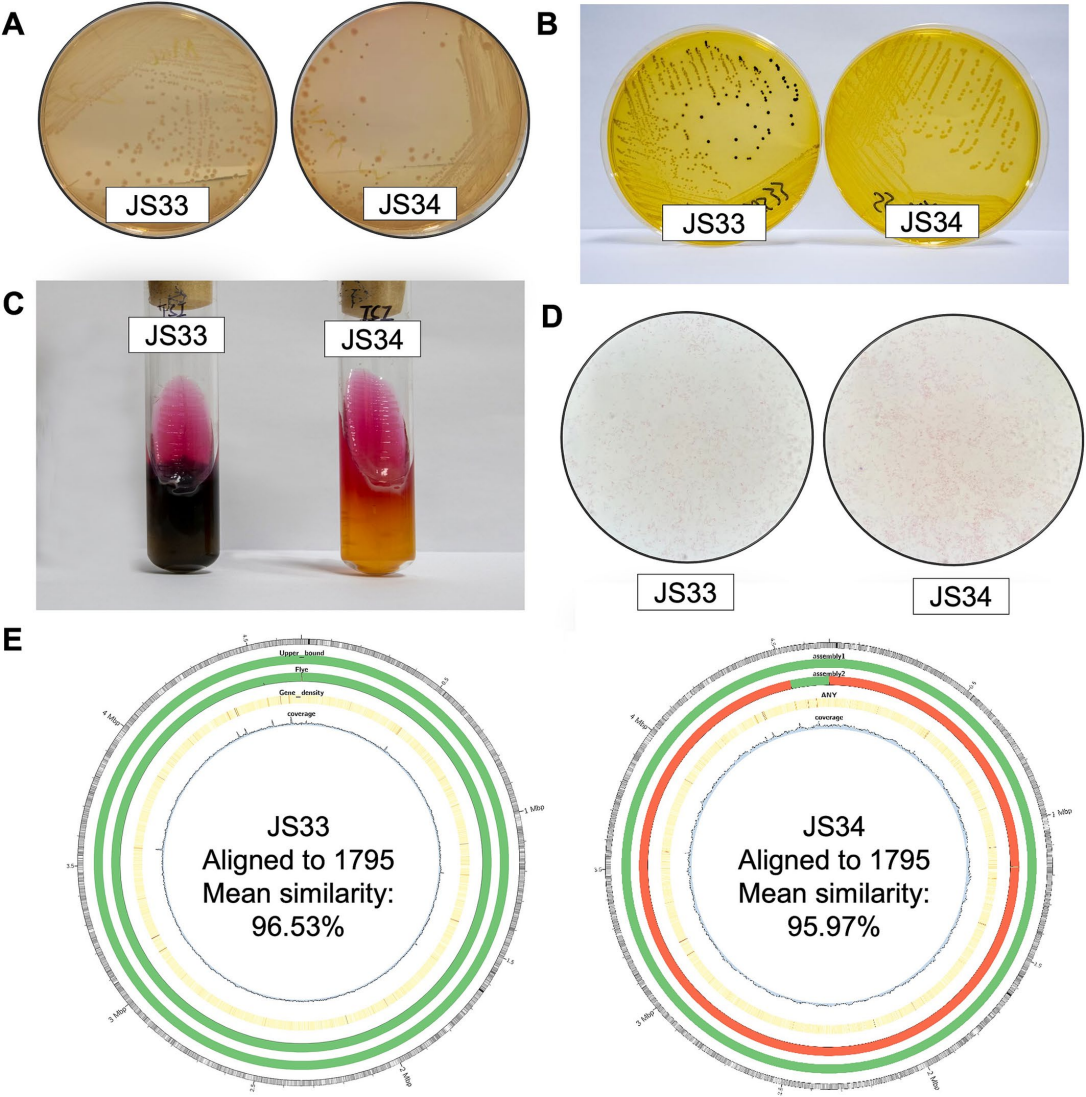
JS33 and JS34 are different in H_2_S generation. **(A–C)** JS33 and JS34 were cultured in MacConkey agar **(A)**, SS agar medium **(B)**, and three-sugar iron agar slant **(C)**. **(D)** The Gram-stained microscopic examination of JS33 and JS34. **(E)** Aligned to CP091611.1 *Salmonella enterica* strain 1795 chromosome, complete genome (from outer to the inner: 1. GC ratio; 2. QUAST gives parametric assembly results; 3. Flye *De Novo* assembly results; 4. Gene density; 5. Depth of sequencing coverage).

Colorless, translucent, and smooth round colonies were observed on MacConkey agar medium (Figure 1A), supporting that JS33 and JS34 belonged to *Salmonella* (Farhoudi Moghaddam et al., 1988). In addition, JS33 and JS34 formed round, moist, smooth, translucent colonies that became lighter in color on the SS agar medium (Figure 1B). However, JS33 was a colony with a black center, which differed from JS34 (Figure 1B). These two bacterial isolates with inconsistent morphology on the SS agar medium were selected and inoculated on the three-sugar iron agar slant (Figure 1C). It showed that both isolates fermented glucose and produced acid and gas but did not ferment lactose and sucrose, as these two gas-produced (+) isolates showed acid (K) on the slant and alkali (A) on the bottom (Figure 1C).

Interestingly, JS33 (K/A++) generated H_2_S (+) (black), while JS34 (K/A + -) did not produce H_2_S (−) (Figure 1C). Gram-stained microscopic examination revealed both isolates as Gram-negative bacilli due to the appearance of a loosely distributed red color (Figure 1D). Gram-negative bacterial identification cards also identified high similarity in JS33 and JS34 except for the production of H_2_S (Table 1, bold text). The serotypes of the two suspected *Salmonella* isolates were 6, 8: r: l, w, which could be identified as *Salmonella enterica* serovar Goldcoast according to the White-Kauffmann-Le Minor antigenic table. The 2nd generation sequencing also identified JS33 and JS34 as *S.* Goldcoast and there were structural variations in the JS34 assembled genome and some of the original reads, compared with the reference genome (Figure 1E).

**Table 1:** Gram-negative testing of JS33 and JS34.

JS33 *Salmonella* group (98% probability)											
APPA	−	ADO	−	PyrA	−	IARL	−	dCEL	−	BGAL	−
**H2S**	**+**	BNAG	−	AGLTp	−	dGLU	+	GGT	−	OFF	+
BGLU	−	dMAL	+	dMAN	+	dMNE	+	BXYL	−	BAlap	−
ProA	−	LIP	−	PLE	−	TyrA	+	URE	−	dSOR	+
SAC	−	dTAG	−	dTRE	+	CIT	+	MNT	−	5KG	+
lLATk	+	AGLU	−	SUCT	+	NAGA	−	AGAL	+	PHOS	+
GlyA	−	ODC	+	LDC	+	IHISa	−	CMT	+	BGUR	−
O129R	+	GGAA	−	lMLTa	−	ELLM	−	lLATa	−		
JS34 *Salmonella* group (97% probability)											
APPA	−	ADO	−	PyrA	−	IARL	−	dCEL	−	BGAL	−
**H2S**	**−**	BNAG	−	AGLTp	−	dGLU	+	GGT	−	OFF	+
BGLU	−	dMAL	+	dMAN	+	dMNE	+	BXYL	−	BAlap	−
ProA	−	LIP	−	PLE	−	TyrA	+	URE	−	dSOR	+
SAC	−	dTAG	−	dTRE	+	CIT	+	MNT	−	5KG	+
lLATk	+	AGLU	−	SUCT	+	NAGA	−	AGAL	+	PHOS	+
GlyA	−	ODC	+	LDC	+	IHISa	−	CMT	+	BGUR	−
O129R	+	GGAA	−	lMLTa	−	ELLM	−	lLATa	−		

“+” represents a positive result, “−” represents a negative result. Bold text highlights the difference in the production of H_2_S.

### 3.2 JS33 and JS34 isolates show multidrug resistance

Based on the latest version of CLSI breakpoints, JS33 and JS34 were evaluated with MDR to ampicillin (AMP), compound sulfamethoxazole (or selectrin, SXT), chloramphenicol (CHL), tetracycline (TET), and streptomycin (STR) (Table 2). They were both intermediate-resistant to ampicillin/sulbactam (AMS), colistin (CT), and polymyxin (BPOL). Besides, JS33 was sensitive to cefazolin (CFZ), while JS34 was intermediate-resistant (Table 2). Both JS33 and JS34 were sensitive to azithromycin (AZM), ciprofloxacin (CIP), nalidixic acid (NAL), and gentamicin (GEN), as well as cefotaxime (CTX), ceftazidime (CAZ), cefoxitin (CFX), imipenem (IPM), amoxicillin/clavulanic acid (AMC), cefuroxime (CXM), cefepime (CPM), ceftazidime-avibactam (CZA), meropenem (MEM), ertapenem (ETP), tigecycline (TGC), and amikacin (AMI).

**Table 2:** Results of drug-susceptibility testing.

Antimicrobial agents	JS33	JS34	MIC (ug/ml)		
			S	I	R
AZM	4(S)	4(S)	≤16/8	/16	≥32
AMP	>64(R)	>64(R)	≤8	16^	≥32
AMS	16(I)	16(I)	≤8/4	16/8^	≥32/16
CIP	0.25(S)	0.25(S)	≤0.25	0.5^	≥1
SXT	>8(R)	>8(R)	≤2/38	-	≥4/76
CHL	>64(R)	>64(R)	≤8	16	≥32
NAL	8(S)	8(S)	≤16	-	≥32
GEN	≤1(S)	≤1(S)	≤2	4^	≥8
TET	>32(R)	>32(R)	≤4	8	≥16
CTX	≤0.25(S)	≤0.25(S)	≤1	2^	≥4
CAZ	≤0.5(S)	1(S)	≤4	8^	≥16
CFX	4(S)	4(S)	≤8	16^	≥32
CFZ	2(S)	4(I)	≤2	4	≥8
IPM	≤0.25(S)	≤0.25(S)	≤1	2^	≥4
CT	0.25(I)	1(I)	-	≤2	≥4
BPOL	0.25(I)	2(I)	-	≤2	≥4
AMC	8(S)	8(S)	≤8/4	16/8^	≥32/16
CXM	8(S)	8(S)	≤8	16^	≥32
CPM	≤1(S)	≤1(S)	≤2	-	≥16
CZA	0.5(S)	0.5(S)	≤8/4	-	≥16/4
MEM	≤0.12(S)	≤0.12(S)	≤1	2^	≥4
ETP	≤0.25(S)	≤0.25(S)	≤0.5	1^	≥2
TGC	0.5(S)	0.5(S)	≤4	8	≥16
AMI	≤4(S)	≤4(S)	≤4	8^	≥16
STR	32(R)	>32(R)	≤16	-	≥32

“^” Drugs with potential to concentrate in urine.

### 3.3 Deep sequencing reveals ten protein-coding genes exclusively expressed in either JS33 or JS34

**Figure 2:**
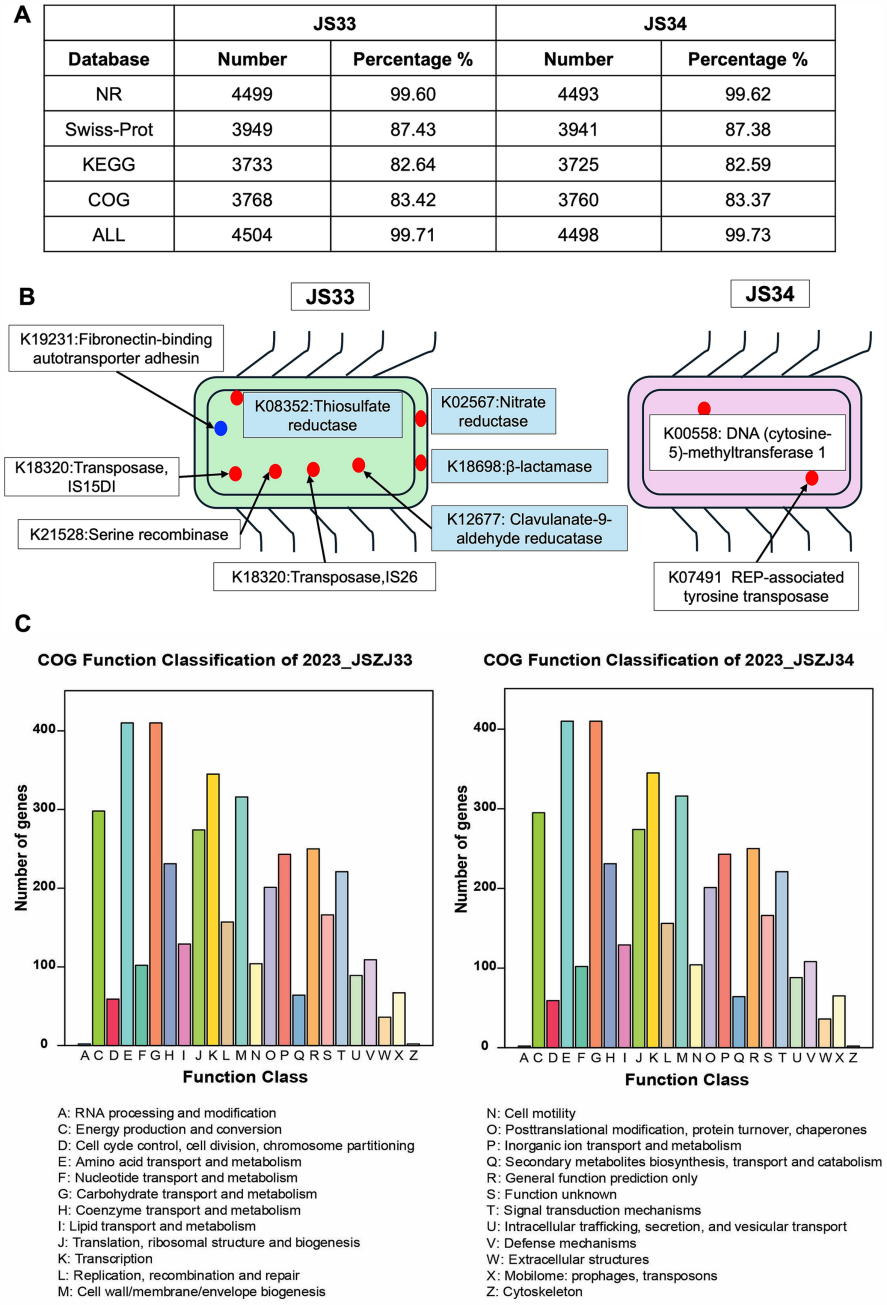
Ten protein-coding genes are exclusively expressed either in JS33 or JS34. **(A)** Annotated sequence number ascribed in NR, Swiss-port, KEGG, and COG in JS33 and JS34, respectively. **(B)** Exclusive protein-encoding genes in JS33 or JS34. The blue point represents proteins with unknown subcellular localization, while the red points represent proteins with specific localization. **(C)** The COG function classification of JS33 and JS34 samples.

To elucidate the genetic background, we extracted DNA samples from purified JS33 and JS34 isolates and subjected them to deep sequencing using GridION (Oxford Nanopore Technology). This process yielded a comparable annotated sequence number in Non-Redundant (NR), Swiss-port, Kyoto Encyclopedia of Genes and Genomes (KEGG), and Clusters of Orthologous Genes (COG) databases in JS33 and JS34, respectively (Figure 2A). Ten protein-coding genes were exclusively expressed in either JS33 or JS34 (Figures 2A,B). Among them, the thiosulfate reductase (K08352), nitrate reductase (K02567), *β*-lactamase (K18698), and clavulanate-9-aldehyde reductase (K12677) were unique to JS33. Other proteins, such as fibronectin-binding autotransporter adhesin (K19231), DNA (cytosine-5)-methyltransferase 1(K00558), and REP-associated tyrosine transposase (K07491), were expressed in both JS33 and JS34, but with different gene numbers (Figure 2B).

Despite the distinct genome, the function classification identified high genetic similarities between JS33 and JS34. The COG function classification was consistent in both isolates, with only minor variations in the number of genes (Figure 2C). Amino acid transport and metabolism, carbohydrate transport and metabolism, transcription, cell wall/membrane/envelope biogenesis, and energy production and conversion were the top five gene-enriched functions, highlighting the shared roles of JS33 and JS34. The KEGG analysis consistently emphasized the roles of JS33 and JS34 in regulating metabolism and participating in genetic information processing (Supplementary Figure S1).

### 3.4 *β*-Lactamase is related to MDR, while the deficiency of thiosulfate reductase inhibits H_2_S production in JS34

**Figure 3:**
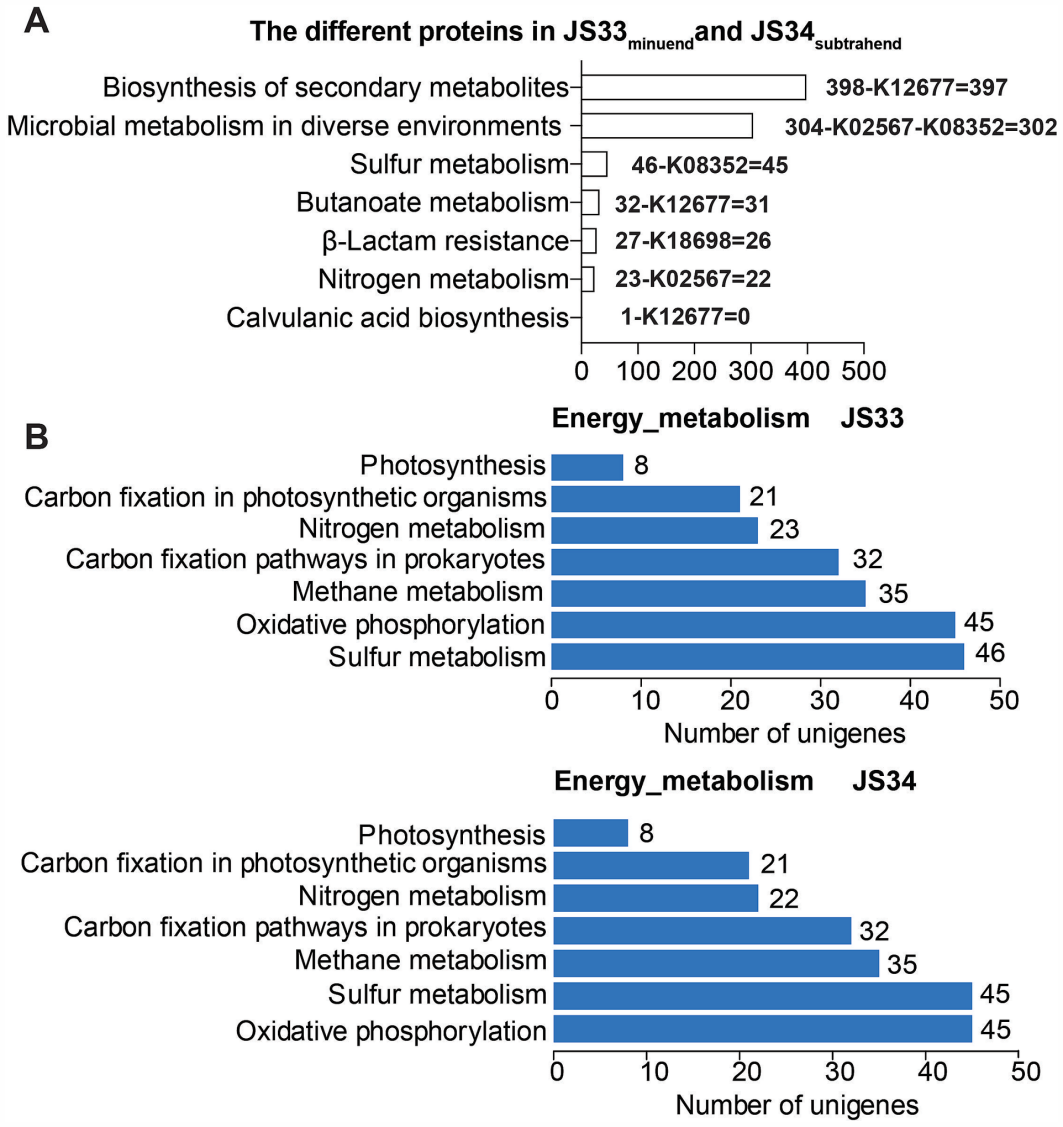
Distinct genome affects drug resistance and H_2_S production. **(A)** The annotated sequence number of JS33 differed from JS34 in KEGG pathways. **(B)** The annotated sequence number of JS33 and JS34 in KEGG energy metabolism pathways.

Deep sequencing also revealed that JS33 and JS34 were closely related to infectious disease and drug resistance, while one more gene was identified in JS33, which encoded β-lactamase class A (Figure 2B; Supplementary Figure S1). β-lactamases are the most common reason resulting in resistance to β-lactam antibiotics in Gram-negative bacteria (Bush and Bradford, 2019). By combing the detailed KEGG classification with gene identification, we found that the exclusively expressed K12677 and K18698 participated in the biosynthesis of secondary metabolites, butanoate metabolism, β-lactam resistance, and clavulanic acid biosynthesis. They were responsible for mild differences in drug response between JS33 and JS34 (Figure 3A). K02567 and K08352 participated in energy metabolism by regulating nitrogen and sulfur metabolism, respectively. Particularly, K08352 played an essential role in H_2_S production (Figure 3B).

**Figure 4:**
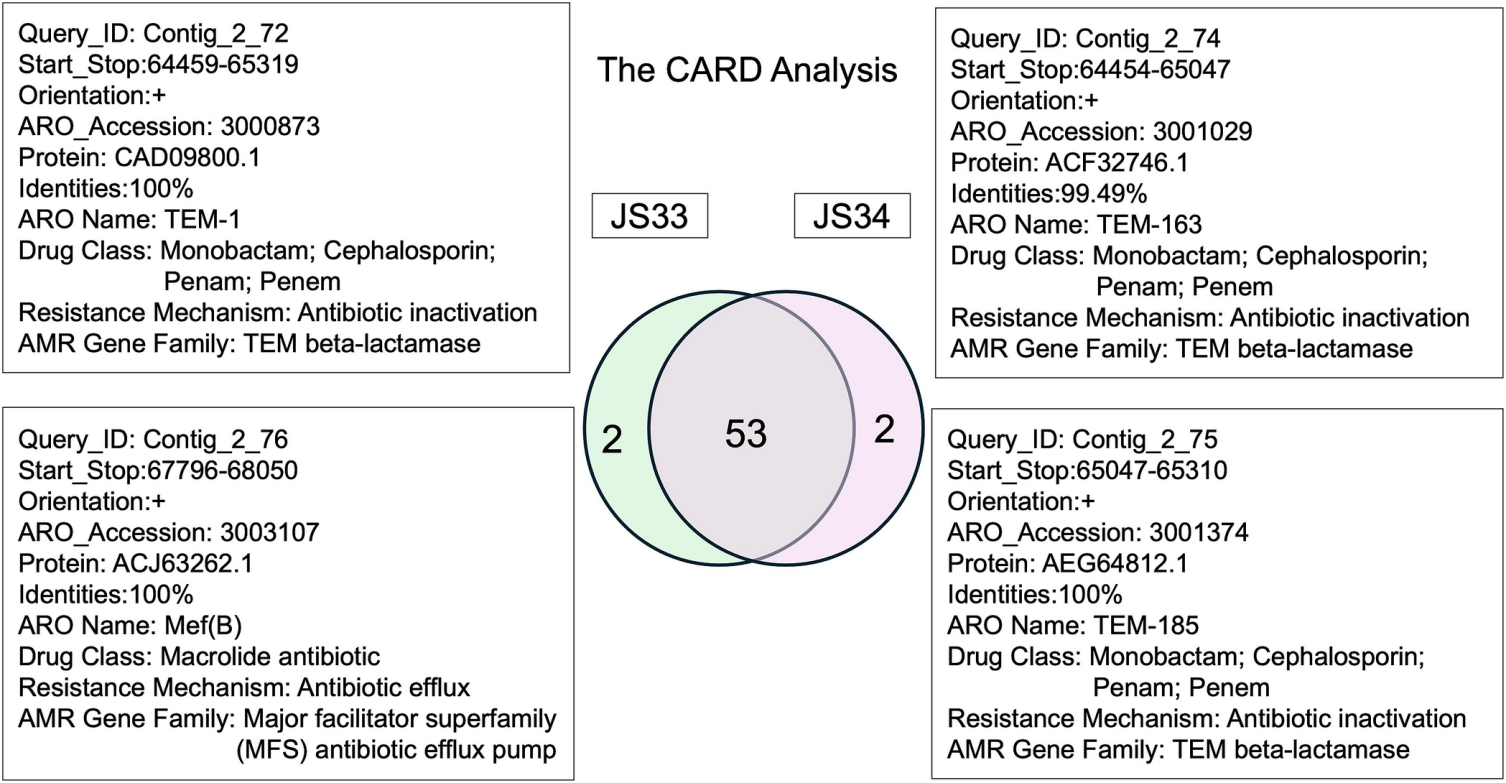
The comprehensive antibiotic resistance database in JS33 and JS34.

Moreover, the resistance gene identifier (RGI) identified 55 resistance genes (42 in config1 and 13 in config2, >50% identities, E-value<0.00001), while 53 ARGs were common in JS33 and JS34, and 45 of 55 ARGs showed more than 90% identities (Figure 4). Based on the analysis of virulence factors in pathogenic bacteria, we found that the gene encoded K19231 in JS33 was linked to the upaH gene, which regulates the AIDA-I type autotransporter protein, a rarely glycosylated protein. The gene encoded K02567 was associated with the nuoG gene and functioned as an anti-apoptosis factor. The gene encoded K08352 was related to narG and was involved in anaerobic respiration. However, the differentially expressed genes did not correlate with bacterial virulence in JS34.

Furthermore, the subcellular localization of secretory proteins in JS33 and JS34 were similar based on PSORTb analysis^1^ (Yu et al., 2010). Most secretory proteins were located at cytoplasmic and fewer were in cytoplasmic membrane, while a few were in periplasmic. The Prophage prediction based on PHAge Search Tool Enhanced Release software (PHASTER) also showed high similarity in contig_1 and relatively less similarity in contig_2 between JS33 and JS34^2^ (Arndt et al., 2016). Genomic island prediction based on Island Viewer and Crispr-Cas prediction based on CRISPR finder^3^ were the same as each other (Couvin et al., 2018). No difference was observed in JS33 and JS34 based on the carbohydrate-active enzymes database.

## 4 Discussion and conclusion

JS33 and JS34 were identified as *Salmonella enterica* serovar Goldcoast with almost identical biological properties. The only remarkable difference between JS33 and JS34 was H_2_S generation based on the three-sugar iron agar slant and Gram-negative bacterial identification. Consistent with the phenotypic observation, deep sequencing also identified high similarity in genetic information. Only ten genes and four proteins were exclusively expressed in JS33 or JS34. There were two different genes relating to antibiotic resistance, i.e., TEM-185 and TEM-163, and three relating to virulence factors, i.e., narG, nuoG, and upaH.

The K18698 represented *β*-lactamase, and K12677 represented clavulanate-9-aldehyde reductase affected resistance to antibiotics in JS33. Specifically, β-lactams are the most widely used antibacterial agents worldwide, while β-lactamases are capable of deacylating β-lactam-derived covalent complexes, representing the most critical resistance mechanism in Gram-negative bacteria (Mora-Ochomogo and Lohans, 2021). However, the presence of β-lactamases did not generate a remarkable impact on the efficacy of antibiotics in JS33 compared with JS34. It was because of the coexistence of clavulanate-9-aldehyde reductase catalyzed the biogenesis of clavulanic acid, an important inhibitor of β-lactamases in JS33 (Docquier and Mangani, 2018). Clinically, β-lactam antibiotics are frequently administered with a β-lactamase inhibitor, such as clavulanic acid, that protects the antibiotic from β-lactamase catalyzed degradation (Huttner et al., 2020).

K02567 represented nitrate reductase, and K08352 represented thiosulfate reductase, which participated in nitrogen metabolism and sulfur metabolism, respectively. Particularly, thiosulfate reductase deficiency resulted in an ultimate inhibition of H_2_S production in JS34. Thiosulfate reductase activity is found in numerous microorganisms, whereas the molecular mechanism of reductive cleavage of thiosulfate is not yet known in bacteria (Le Faou et al., 1990). The comparison between JS33 and JS34 could help to illustrate the role of thiosulfate reductase in H_2_S production in bacteria. No other significant difference was observed between JS33 and JS34 in the subcellular localization of secretory protein, the Prophage prediction, or genomic island prediction.

However, JS33 and JS34 differed from previously reported pathogenic *S*. Goldcoast in Zhejiang province in that they did not induce any clinical symptoms. There were three cases of *S.* Goldcoast that were collected in Zhejiang province, and two of them came from human hosts (Wang et al., 2023a, 2023b). The XXB830 (2015, 1-year-old, Female) was extracted from human feces which caused the gastrointestinal infection, while XXB1582 (2017, 67-year-old, Male) was extracted from the blood which caused the extra-intestinal infection. By downloading public files from the Chinese Local Salmonella Genome DataBase version 2, we compared previously reported cases with JS33 and JS34 (Wang et al., 2023b). Only 1 of 1,323 genes relating to virulence factors were different between XXB830 and XXB1582, but 169 of 1,323 genes relating to virulence factors disappeared in JS33 and JS34. Of course, the difference in accuracy between the second-generation sequencing and the third-generation sequencing may cause some errors in data analysis. On the contrary, JS33 showed the exact same antibiotic-resistant genes compared with XXB830. Moreover, the annotated sequence number ascribed in NR, Swiss-port, KEGG, and COG, as well as function classification, was similar in all *S*. Goldcoast samples.

Identifying new *S*. Goldcoast strains was consistent with previously estimated increased tendency and alerted a prevalence of *S*. Goldcoast with MDR in southeast China. Besides, due to the extensive similarities and specific differences between JS33 and JS34, they could perform as model strains to help us understand microbial antibiotic resistance and study microbial H_2_S. However, the conclusions of this study are limited by the small sample size. As *S*. Goldcoast was the 16th serotype of *S. enterica* in China, accounting for 0.91% of geographical distribution, a comprehensive monitor of *S*. Goldcoast was needed but has not drawn much attention from the public (Wang et al., 2023a). The surveillance of non-pathogenic but multidrug-resistant isolates from healthy populations was especially unsatisfying and urgently needed.

## Data Availability

The genome sequence of Salmonella enterica serovar Goldcoast strains JS33 and JS34 (Biosample ID: 2023_JSZJ_022 and 2023_JSZJ_023) can be accessed at DDBJ/ENA/GenBank under the accession number PQ613620. The R script and analysis report was deposited in GitHub (https://github.com) public repository, zhouli3-wz/JS33-and JS34.

## Footnotes

^1^ http://www.psort.org

^2^ https://phaster.ca

^3^ https://crisprcas.i2bc.paris-saclay.fr/CrisprCasFinder/Index

## References

[ref1] Aleksandrowicz A. Carolak E. Dutkiewicz A. Błachut A. Waszczuk W. Grzymajlo K. (2023). Better together-Salmonella biofilm-associated antibiotic resistance. Gut Microbes 15:2229937. doi: 10.1080/19490976.2023.2229937, PMID: 37401756 PMC10321201

[ref2] Arndt D. Grant J. R. Marcu A. Sajed T. Pon A. Liang Y.. (2016). PHASTER: a better, faster version of the PHAST phage search tool. Nucleic Acids Res. 44, W16–W21. doi: 10.1093/nar/gkw387, PMID: 27141966 PMC4987931

[ref3] Bush K. Bradford P. A. (2019). Interplay between β-lactamases and new β-lactamase inhibitors. Nat. Rev. Microbiol. 17, 295–306. doi: 10.1038/s41579-019-0159-8, PMID: 30837684

[ref4] Centers for Disease Control and Prevention (CDC). National antimicrobial resistance monitoring system NARMS 2015 human isolates surveillance report. Atlanta, GA: (2018). Available at: https://www.cdc.gov/narms/pdf/2015-NARMS-Annual-Report-cleared_508.pdf

[ref5] Clinical and Laboratory Standards Institute (CLSI). (2023). M100 performance standards for antimicrobial susceptibility testing. 28 ed. Wayne, PA. Available at: https://community.clsi.org/media/1930/m100ed28_sample.pdf

[ref6] Couvin D. Bernheim A. Toffano-Nioche C. Touchon M. Michalik J. Néron B.. (2018). CRISPRCasFinder, an update of CRISRFinder, includes a portable version, enhanced performance and integrates search for Cas proteins. Nucleic Acids Res. 46, W246–w251. doi: 10.1093/nar/gky425, PMID: 29790974 PMC6030898

[ref7] Darby E. M. Trampari E. Siasat P. Gaya M. S. Alav I. Webber M. A.. (2023). Molecular mechanisms of antibiotic resistance revisited. Nat. Rev. Microbiol. 21, 280–295. doi: 10.1038/s41579-022-00820-y, PMID: 36411397

[ref8] Docquier J.-D. Mangani S. (2018). An update on β-lactamase inhibitor discovery and development. Drug Resist. Updat. 36, 13–29. doi: 10.1016/j.drup.2017.11.002, PMID: 29499835

[ref9] Farhoudi Moghaddam A. A. Katouli M. Parsi M. (1988). Comparison of Teknaf enteric agar and MacConkey/salmonella-shigella agar in evaluation of Shigella infection. Lancet 1, 1165–1166. doi: 10.1016/S0140-6736(88)91979-4, PMID: 2896983

[ref10] Gal-Mor O. Boyle E. C. Grassl G. A. (2014). Same species, different diseases: how and why typhoidal and non-typhoidal *Salmonella enterica* serovars differ. Front. Microbiol. 5:391. doi: 10.3389/fmicb.2014.00391, PMID: 25136336 PMC4120697

[ref11] Gaurav A. Bakht P. Saini M. Pandey S. Pathania R. (2023). Role of bacterial efflux pumps in antibiotic resistance, virulence, and strategies to discover novel efflux pump inhibitors. Microbiology (Reading) 169:1333. doi: 10.1099/mic.0.001333, PMID: 37224055 PMC10268834

[ref12] Han S. Li Y. Gao H. (2022). Generation and physiology of hydrogen sulfide and reactive sulfur species in Bacteria. Antioxidants (Basel) 11, 2487–2512. doi: 10.3390/antiox11122487, PMID: 36552695 PMC9774590

[ref13] Hoffmann S. Batz M. B. Morris J. G. Jr. (2012). Annual cost of illness and quality-adjusted life year losses in the United States due to 14 foodborne pathogens. J. Food Prot. 75, 1292–1302. doi: 10.4315/0362-028X.JFP-11-417, PMID: 22980013

[ref14] Huttner A. Bielicki J. Clements M. N. Frimodt-Møller N. Muller A. E. Paccaud J. P.. (2020). Oral amoxicillin and amoxicillin-clavulanic acid: properties, indications and usage. Clin. Microbiol. Infect. 26, 871–879. doi: 10.1016/j.cmi.2019.11.028, PMID: 31811919

[ref15] Lamichhane B. Mawad A. M. M. Saleh M. Kelley W. G. Harrington P. J. Lovestad C. W.. (2024). Salmonellosis: an overview of epidemiology, pathogenesis, and innovative approaches to mitigate the antimicrobial resistant infections. Antibiotics (Basel) 13, 76–126. doi: 10.3390/antibiotics13010076 38247636 PMC10812683

[ref16] Le Faou A. Rajagopal B. S. Daniels L. Fauque G. (1990). Thiosulfate, polythionates and elemental sulfur assimilation and reduction in the bacterial world. FEMS Microbiol. Rev. 6, 351–381. doi: 10.1016/0378-1097(90)90688-M, PMID: 2123394

[ref17] Manesh A. Meltzer E. Jin C. Britto C. Deodhar D. Radha S.. (2021). Typhoid and paratyphoid fever: a clinical seminar. J. Travel Med. 28, 1–13. doi: 10.1093/jtm/taab012, PMID: 33550411

[ref18] Mora-Ochomogo M. Lohans C. T. (2021). β-Lactam antibiotic targets and resistance mechanisms: from covalent inhibitors to substrates. RSC Med. Chem. 12, 1623–1639. doi: 10.1039/D1MD00200G, PMID: 34778765 PMC8528271

[ref19] Smith S. I. Seriki A. Ajayi A. (2016). Typhoidal and non-typhoidal Salmonella infections in Africa. Eur. J. Clin. Microbiol. Infect. Dis. 35, 1913–1922. doi: 10.1007/s10096-016-2760-3, PMID: 27562406

[ref20] Wang Y. Liu Y. Lyu N. Li Z. Ma S. Cao D.. (2023a). The temporal dynamics of antimicrobial-resistant Salmonella enterica and predominant serovars in China. Natl. Sci. Rev. 10, 1–17. doi: 10.1093/nsr/nwac269, PMID: 37035020 PMC10076184

[ref21] Wang Y. Xu X. Zhu B. Lyu N. Liu Y. Ma S.. (2023b). Genomic analysis of almost 8,000 Salmonella genomes reveals drivers and landscape of antimicrobial resistance in China. Microbiol. Spectr 11, e02080–e02023. doi: 10.1128/spectrum.02080-23 37787535 PMC10714754

[ref22] Yu N. Y. Wagner J. R. Laird M. R. Melli G. Rey S. Lo R.. (2010). PSORTb 3.0: improved protein subcellular localization prediction with refined localization subcategories and predictive capabilities for all prokaryotes. Bioinformatics 26, 1608–1615. doi: 10.1093/bioinformatics/btq249, PMID: 20472543 PMC2887053

