# Supplementary material for: Genomic identification of a pair of multidrug-resistant but non-pathogenic Salmonella enterica serovar Goldcoast isolates in southeast China
Source: Front Microbiol. 2025 Feb 26;16:1540843. doi: 10.3389/fmicb.2025.1540843 (PMC11897504; doi:10.3389/fmicb.2025.1540843)
Supplement: Supplementary file 1 [file Table_1.docx]

Supplementary Material

# Supplementary Figures


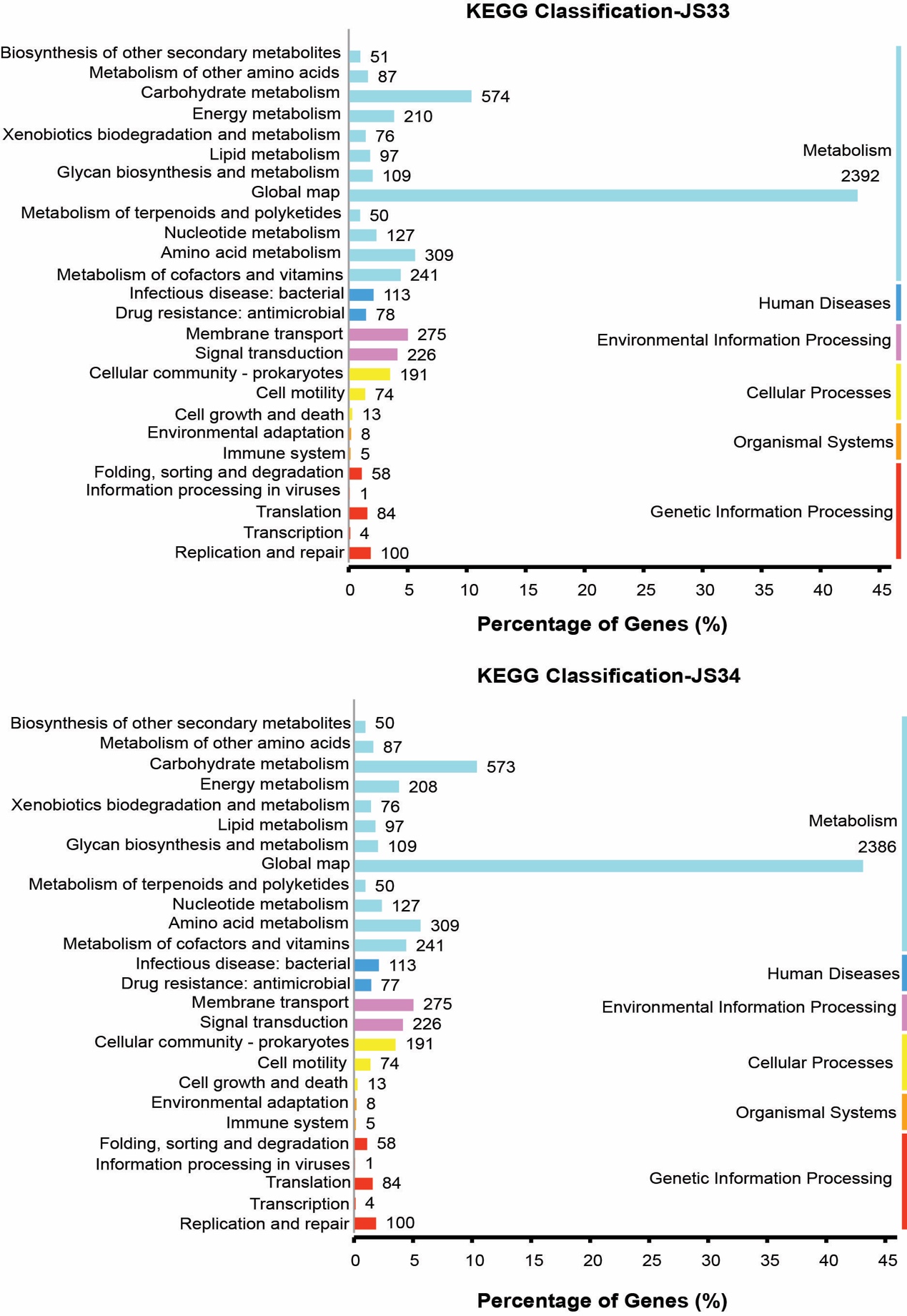


**Supplementary Figure 1.** The KEGG classification of genes expressed in JS33 and JS34.
